# *Veillonella parvula* as a Causative Agent of Discitis: Insights from a Clinical Case and Literature Overview

**DOI:** 10.3390/antibiotics14090854

**Published:** 2025-08-24

**Authors:** Giulio D’Agati, Lorena Mignone, Antonella Bartolone, Giuseppa Sciortino, Teresa Maria Assunta Fasciana, Cinzia Calà, Silvia Bonura, Francesco Carini, Luca Pipitò, Antonio Cascio

**Affiliations:** 1 Department of Health Promotion, Mother and Child Care, Internal Medicine and Medical Specialties “G D’Alessandro”, University of Palermo, 90127 Palermo, Italy; giulio.dagati@community.unipa.it (G.D.); lorena.mignone@community.unipa.it (L.M.); antonella.bartolone@community.unipa.it (A.B.); teresa.fasciana@unipa.it (T.M.A.F.); cinzia.cala@unipa.it (C.C.); luca.pipito@community.unipa.it (L.P.); 2 Infectious and Tropical Disease Unit, AOU Policlinico “P. Giaccone”, 90127 Palermo, Italy; silvia.bonura@policlinico.pa.it; 3 Microbiology and Virology Unit, AOU Policlinico “P. Giaccone”, 90127 Palermo, Italy; giuseppa.sciortino@policlinico.pa.it; 4 Biomedicine, Neurosciences and Advanced Diagnostics BIND, School of Medicine, University of Palermo, 90133 Palermo, Italy; francesco.carini@unipa.it

**Keywords:** *Veillonella* spp., *Veillonella parvula*, discitis, spondylodiscitis, vertebral osteomyelitis

## Abstract

Background/Objectives: *Veillonella* species are Gram-negative, non-motile, non-fermentative, obligate anaerobic cocci. They are typically considered commensals of the oral cavity, respiratory tract, genitourinary tract, and gastrointestinal tract. It may be a rare cause of dental infections and discitis/spondylodiscitis. Methods: We report the case of an 80-year-old patient diagnosed with discitis caused by *Veillonella parvula*, isolated from blood. In addition, we performed a comprehensive literature review summarizing all reported cases of discitis or spondylodiscitis caused by *Veillonella* species. Results: In our case, antimicrobial susceptibility testing was performed using the Kirby–Bauer disc diffusion method. Based on the results, the patient was treated with amoxicillin/clavulanate, which led to a favourable clinical outcome. A review of the literature revealed that, to date, only 14 cases of spondylodiscitis or discitis caused by *Veillonella* spp. have been reported. Potential risk factors for *Veillonella* spp. bacteremia were identified in only 9 cases. The most commonly affected site was the lumbar or lumbosacral spine. Magnetic resonance imaging was consistently regarded as the diagnostic gold standard. Most patients presented with localized pain. The overall therapeutic approach generally consisted of an initial course of intravenous antibiotics, typically ceftriaxone administered either as monotherapy or in combination with metronidazole, followed by an oral regimen with amoxicillin/clavulanate, given alone or alongside metronidazole. Conclusions: Spondylodiscitis due to *V. parvula* remains extremely rare. Although antimicrobial susceptibility patterns remain heterogeneous, beta-lactams, particularly amoxicillin/clavulanate, appear effective in most cases, and treatment regimens typically involve an initial intravenous phase followed by oral therapy.

## 1. Introduction

*Veillonella* species are Gram-negative, non-motile, non-fermentative, and obligate anaerobic cocci. The genus *Veillonella* was initially described by Veillon and Zuber in 1898, with its name later proposed by Prevot in 1933, and it is currently classified based on DNA homology [1]. The genus includes six recognized species: *V. parvula*, *V. atypica*, *V. alcalescens*, *V. dispar*, *Acidaminococcus fermentans*, and *Megasphaera elsdenii*, and they are generally considered commensals of the oral cavity (approximately 5%), respiratory tract, urogenital tract, and gastrointestinal tract [2,3]. *Veillonella* spp. metabolize lactic acid produced from sugar fermentation as a carbon source, contributing to biofilm formation in the oral environment [4]. These microorganisms were rarely associated with infections, and risk factors include diabetes mellitus, intravenous drug use, malignancies, immunodeficiency states, renal failure, recent gastrointestinal procedures, endoscopic biopsies, open fractures, post-procedural esophageal perforation, as well as various surgeries or invasive interventions [2,5,6]. *V. parvula* is the most frequently isolated species, with GenBank accession number AF439640 [2,5]. Previous reported infections due to *V. parvula* include cases of dental infections [7], discitis/spondylodiscitis [2,8], endocarditis [9], non-vertebral osteomyelitis [10,11], meningitis [12], hepatic and pulmonary infections [13], bacteremia [14,15], decubitus ulcers [15], prosthetic joint infections [16,17], and fatal sepsis [18]. A review by Hirai et al. identified 31 cases of *Veillonella* spp. infection in humans from 1976 to October 2015 [19]. Therapeutic guidelines for *Veillonella* spp. infections are not well established due to the paucity of reported cases. Penicillin remains the treatment of choice, and where resistance to penicillin G exists, susceptibility to amoxicillin/clavulanic acid is generally retained. Alternative agents include cephalosporins, clindamycin, chloramphenicol, and metronidazole, while resistance is common to tetracyclines, vancomycin, aminoglycosides, ciprofloxacin, and intermediate susceptibility to erythromycin is noted [3,20]. Here, we report a case of invasive infections caused by *V. parvula*, presenting as spondylodiscitis, and provide a comprehensive literature review.

## 2. Case Report

### 2.1. Medical History

An 80-year-old man was admitted to the Infectious and Tropical Diseases Unit at the University Hospital “P. Giaccone” in Palermo, Italy, in 2024 with a diagnosis of lumbar spondylodiscitis. His past medical history included recently diagnosed epilepsy, ischemic heart disease treated with placement of two coronary stents, arterial hypertension, chronic cerebrovascular disease, hypothyroidism following thyroidectomy, and permanent dental prosthesis for edentulism related to age and previous carious foci.

### 2.2. Previous Spinal Surgeries

**Figure 1:**
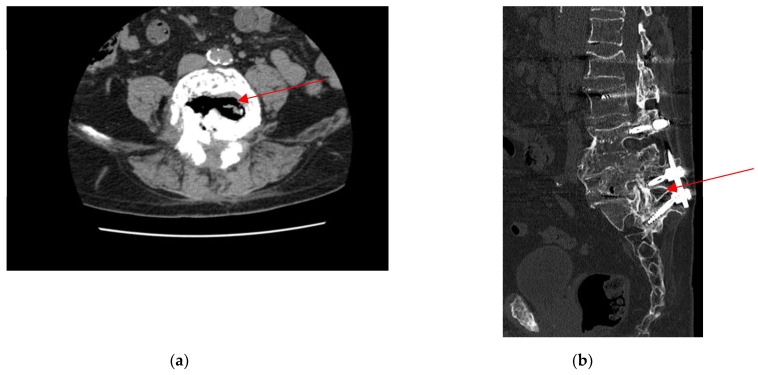
(**a**) Collapse of the L4 vertebral body, which appears fragmented with retropulsion of the posterior wall. Gas degeneration of the interposed L4–L5 disc (red arrow); (**b**) Post-surgical changes from L4 vertebral body collapse, treated with transpedicular plates and screws, currently well-positioned from L1 to S1. Gas degeneration of the interposed L4–L5 disc (red arrow). Surgical drains with distal ends adjacent to the spinous processes of L1 laterally, and paravertebral air bubbles between L1 and S1.

Due to recurrent and multifocal disc herniation, he had undergone two spinal stabilization surgeries with placement of rods 31 and 23 years prior, respectively. An additional spinal stabilization procedure with laminectomy was performed four years before admission, during which bone and disc fragments were collected and submitted for microbiological analysis. Five days later, culture of a disc specimen grew *Enterococcus faecium*, establishing the diagnosis of lumbar spondylodiscitis (L4–L5) complicated by vertebral body collapse, stenosis of the corresponding spinal canal, and involvement of the dural sac. The patient was subsequently treated with vancomycin (Figure 1).

**Figure 2:**
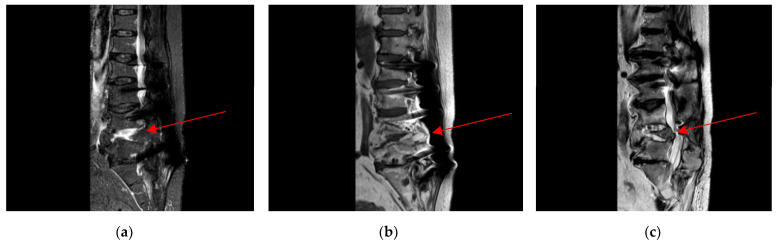
MRI scans in various sequences revealed discal alterations, specifically a hyperintense signal at the intervertebral discs from L3 to L5 on T2-weighted sequences, without evidence of involvement of the adjacent vertebral bodies or surrounding soft tissues (red arrows). Progressive fusion was observed at the residual L4–L5 disc space, associated with right-sided neuroforaminal stenosis. (**a**) Lumbosacral magnetic resonance imaging using STIR sequences. (**b**) Lumbosacral magnetic resonance imaging using T1-weighted sequences. (**c**) Lumbosacral magnetic resonance imaging using T2-weighted sequences.

For approximately eight weeks before admission, the patient had been experiencing debilitating right-sided neuropathic metatarsalgia, supported by radiographic evidence of degenerative joint disease affecting the first metatarsophalangeal joint of the right foot, and refractory to both corticosteroid infiltrations and oral anti-inflammatory therapy. Upon the recommendation of his orthopedic specialist, a contrast-enhanced lumbosacral MRI was performed three days before admission, revealing a hyperintense signal at the L3–L5 intervertebral discs, without evidence of involvement of the adjacent vertebral bodies or surrounding soft tissues. The scan also showed a tendency toward fusion of the residual L4–L5 disc space and associated neuroforaminal stenosis. These findings were suggestive of inflammatory changes that, in the context of the patient’s clinical history, could also be consistent with recurrent spondylodiscitis (Figure 2).

### 2.3. Admission to Infectious Diseases Unit

The patient was admitted to our department for further management. Upon admission, the patient was in fair general clinical condition. He was afebrile and eupneic in room air. He ambulated with the aid of a walker and showed poor compliance with the spinal brace prescribed by the orthopedic specialist. Physical examination was unremarkable, but oral examination revealed the presence of a permanent dental prosthesis in a worn condition. The patient reported persistent pain despite home analgesic therapy, associated with a deficit of dorsiflexion and plantarflexion and paresthesia in the right metatarsal region, exacerbated even by light palpation, in the absence of local signs of inflammation. This presentation was initially consistent with a presumptive diagnosis of radiculopathy. The surgical scar over the lumbar spine appeared dry and in excellent condition. Blood tests revealed a white blood cell (WBC) count of 7080/mm^3^ (normal 4000–11,000/mm^3^) with a normal differential, hemoglobin (Hb) level of 13 g/dL (normal 12–18 g/dL), platelet count (PLT) of 281,000/mm^3^ (150,000–450,000/mm^3^), and C-reactive protein (CRP) level of 5.78 mg/L (normal <5 mg/L).

### 2.4. Neurosurgery Evaluation

**Figure 3:**
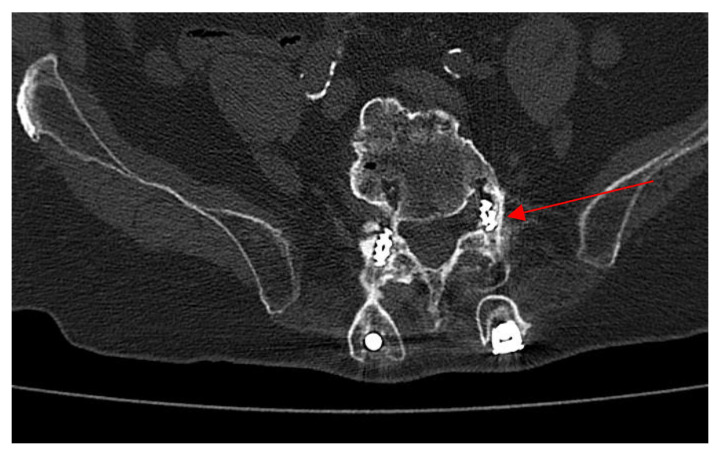
Lumbosacral computed tomography scan, the left L5 pedicle screw obliterates the ipsilateral preforaminal recess (red arrow).

Upon the neurosurgical team’s recommendation, a contrast-enhanced lumbosacral CT scan was performed to reassess the stabilization implant. The CT scan (Figure 3) revealed that the left L5 pedicle screw obliterated the ipsilateral preforaminal recess. Diffuse alterations in the osseous structure of the vertebral bodies were observed in the examined segment, with marked neo-ossification and osteophytosis forming bony bridges, particularly between L4 and L5. These changes were also appreciable in the posterior elements and contributed to the narrowing of both the spinal canal and the intervertebral foramina. Minimal fluid infiltration of the paravertebral soft tissues was noted between L3 and L5. A neurosurgical indication was given for the patient to wear a lumbar bracing orthosis while in the upright (standing) position.

### 2.5. Microbiological Analysis

Given the patient’s clinical stability, empirical antibiotic therapy was not initiated. Due to the potential risks associated with performing spinal biopsies in a patient with a history of prior surgeries in the same area, we opted to obtain blood cultures, even in the absence of fever. Blood cultures were obtained from a peripheral vein under aseptic conditions for microbiological analysis (three sets: aerobic, anaerobic, and fungal). Blood culture vials were incubated in the automated blood culture system BactecTM (BD Diagnostics, Le Pont de Claies, France). After 4 days, growth was detected on an anaerobic blood culture vial. Translucent small colonies grew after seeding in a blood agar plate incubated at 37 °C in CO_2_ for 48 h. Gram-negative cocci were identified as *Veillonella parvula* by 16S rDNA standard analysis with whole-cell MALDI-TOF^®^ MS-based, with a score of 2.15. We also obtained a drug resistance profile using the agar disc diffusion method (Kirby–Bauer susceptibility testing) on Mueller–Hinton agar (Bio-Rad, Marnes-la-Coquette, France) (Table 1). The analysis revealed a large inhibition zone for ampicillin/sulbactam and a reduced inhibition zone for penicillin G, findings that were pivotal in guiding the choice of targeted antibiotic therapy.

### 2.6. Treatment and Follow-Up

While awaiting antimicrobial susceptibility testing, intravenous antibiotic therapy with amoxicillin/clavulanate (1000 mg/200 mg) every 8 h was initiated. The regimen was subsequently confirmed based on susceptibility to ampicillin/sulbactam. The patient received two weeks of intravenous therapy during hospitalization. He was discharged after a three-week hospital stay with instructions to continue oral therapy with amoxicillin/clavulanate (875 mg/125 mg) every 8 h for an additional four weeks. The patient was monitored throughout the entire course of home-based therapy and up to three months after its completion, showing clinical improvement and experienced no laboratory deterioration (improvement in foot pain and without any increase in inflammatory markers).

## 3. Literature Review and Discussion

In the present case, we specifically considered the considerable time elapsed since the previous diagnosis of spondylodiscitis, the complete healing of the surgical wound at the lumbar spine, and the fact that the current presentation was consistent with discitis without vertebral body involvement. For these reasons, we regarded the episode as a new infection. The isolation of *V. parvula* from blood cultures, despite the inherent growth challenges of anaerobic organisms, and the absence of other microorganisms in culture further supported the diagnosis of a new infectious episode rather than a recurrence of *E. faecium* spondylodiscitis.

We conducted a PubMed search using the terms ‘*Veillonella* spp.’, ‘*Veillonella parvula*’, ‘spondylodiscitis’, ‘discitis’, ‘vertebral/spinal osteomyelitis’, and ‘spinal infection’ to identify all reported cases of spondylodiscitis involving *Veillonella* species. We excluded articles not written in English and those in which *Veillonella* was not clearly identified as the infectious agent or as a primary or co-responsible pathogen for the clinical presentation. We also excluded studies lacking adequate clinical information on the patients, such as age, sex, site of infection, and comorbidities. To date, only 14 cases of spondilodiscitis/discitis caused by *Veillonella* spp. have been documented in the literature [Table 2]. The retrieved cases consisted of 11 male and 3 female patients (M:F = 3.7:1), with a mean age of 60 years. In 12 cases (86%), the diagnosis was spondylodiscitis, while in 2 cases (14%) it was discitis. In 1 case (7%), additional sites of infection included the tibia, tricuspid valve, and mitral valve. The most common localization was lumbar or lumbosacral (11/14, 79%), as in our case, followed by thoracolumbar involvement in 2 cases (14%), and cervical involvement in 1 case (7%). MRI was considered the gold standard for diagnosis, and it also proved to be decisive in establishing the diagnosis. Most patients experienced pain, classified as low back pain (LBP) or cervical pain, depending on the anatomical site involved, with a mean symptom duration of approximately 6 weeks. In 3 cases (21%), pain was associated with febrile episodes, with an average duration of approximately 3 weeks. In only 9 cases (64%), potential risk factors for *Veillonella* spp./*V. parvula* bacteremia were identified. Underlying oral cavity diseases were reported in 4 cases (29%), a history of spinal surgery in 2 cases (14%), rheumatoid arthritis in 1 case (7%), and a history of both small bowel and rectal biopsies in 1 case (7%). In 1 case (7%), the patient had undergone bilateral sinusotomy and turbinectomy for carcinoma. *V. parvula* was identified in 9 cases (64%). Peripheral blood samples were collected in 11 cases (78%), but blood cultures were positive in only 5 of them (35%). In 2 cases (14%), the microorganism was identified through PCR analysis. Tissue samples (from vertebrae, intervertebral disc, or paravertebral abscess collections) were obtained in 13 cases (93%). Tissue cultures were positive in 11 of these (79%), and PCR confirmed the pathogen in 1 additional case (7%). Data on antibiotic susceptibility and resistance were available for only 5 cases (36%). In 4 cases (29%), susceptibility to penicillins was documented; however, in 1 of these (7%), there was resistance to penicillin G, and in another case (7%), the strain showed ‘increased exposure’ susceptibility to penicillin G. Therapeutic regimen was not reported in 1 case. In our case, microbiological susceptibility testing was performed using the Kirby–Bauer disc diffusion method, which revealed a small inhibition zone for penicillin G and a larger inhibition zone for ampicillin/sulbactam. These findings supported the choice of amoxicillin/clavulanate for patient treatment. Excluding the patient with concomitant infective endocarditis, the mean duration of therapy was 7 weeks. The general treatment approach consisted of initial intravenous therapy during hospitalization, followed by oral or intramuscular antibiotic therapy at home. This sequential strategy was adopted in 8 cases (57%). In this treatment sequence, intravenous therapy generally consisted of ceftriaxone, administered either as monotherapy (2 cases, 14%) or in combination with metronidazole (2 cases, 14%). Alternative intravenous regimens included amoxicillin (1 case, 7%) and amoxicillin/clavulanate (1 case, 7%). This was followed by oral therapy with amoxicillin/clavulanate, administered either as monotherapy (4 cases, 29%) or in combination with metronidazole (1 case, 7%) or with amoxicillin alone (1 case, 7%). In 2 cases, patients received penicillin G, initially intravenously and subsequently via intramuscular administration. Three patients received the entire course of antibiotic therapy during hospitalization via intravenous administration. This included ampicillin in 1 patient (7%), ceftriaxone as monotherapy in 2 patients (14%), and ceftriaxone combined with metronidazole in 1 patient (7%). One patient (7%) received targeted therapy exclusively via the oral route, with amoxicillin. Surgical intervention was performed in 5 cases (36%). Complete clinical recovery was achieved in 12 patients (86%); the remaining 2 patients were lost to follow-up.

## 4. Conclusions

Spondylodiscitis due to *V. parvula* remains extremely rare, with only 14 previously reported cases to date. Risk factors include prior spinal surgery, dental disease, and systemic comorbidities. Although antimicrobial susceptibility patterns remain heterogeneous, beta-lactams, particularly amoxicillin/clavulanate, appear effective in most cases, and treatment regimens typically involve an initial intravenous phase followed by oral therapy. Early recognition, appropriate microbiological workup, and tailored antibiotic treatment are essential for favourable clinical outcomes. Increased awareness of *Veillonella* spp. as potential pathogens, especially in patients with compatible risk profiles, may improve the detection and management of these uncommon but clinically significant infections.

## Figures and Tables

**Table 1:** Drug resistance profile of *V. parvula* by agar disc diffusion method (Kirby–Bauer susceptibility testing).

Antibiotics	Zone of Inhibition Diameter
Ampicillin/Sulbactam	30.0
Ciprofloxacin	18.0
Gentamicin	18.0
Penicillin G	14.0
Vancomycin	15.0

**Table 2:** Summary of previous cases of Spondylodiscitis caused by *Veillonella* spp.

Author	Age (Year)/Sex	Symptoms (Weeks)	Risk Factors	Discitis/Spondylodiscitis	Other Sites of Infection	(1) Blood and (2) Bone/Disc Cultures	Susceptibility	Therapy (Weeks)	Surgery	Outcome
**(1)**	35/F	LPB (20 w)	Chronic pulpitis and periapical cyst	Spondylodiscitis L4-L5	No	(1) Negative (2) *V. parvula*	S: Amoxicillin Cefotaxime Rifampicin Fluoroquinolones Clindamycin Metronidazole. R: Amikacin Macrolides	Amoxicillin PO (6 w)	No	Cure
**(2)**	76/F	Thoracolumbar junction pain (12 w)	No	Spondylodiscitis L1-L2	No	(1) Negative (2) *Veillonella* spp.	Not reported	Ceftriaxone IV +metronidazolo IV (4 w), then amoxicillina/ clavulanic acid PO + metronidazolo PO (6 w)	Yes	Cure
**(3)**	55/M	LPB + Fever (1 w)	Small bowel biopsy and a rectal biopsy	Spondylodiscitis L3-L4-L5	No	(1) Negative (2) Negative *V. parvula* confirmed by PCR rRNA 16S	Not performed	Ceftriaxone IV (6 w), then amoxicillin/ clavulanic acid PO (6 w)	No	Cure
**(4)**	27/M	LPB (3 w)	No	Discitis L4-L5	No	(1) Negative (2) *Veillonela* spp.	Not reported	Amoxicillin IV (3 w), then amoxicillin PO (8 weeks)	No	Lost during the follow-up
**(5)**	31/M	Odynophagia + Neck pain + Fever (8 w)	Cervical fracture and surgical procedure	Spondylodiscitis C4-C5	No	(1) Not reported (2) *Veillonella* spp. and *Streptococcus viridans* group in the abscess fluid culture	Not reported	Penicillin G IV/IM (6 w)	Yes	Cure
**(6)**	79/M	LBP (4 w)	No	Spondylodiscitis L3-L4	No	(1) Negative *V. parvula* confirmed by PCR rRNA 16S (2) Negative	Not reported	Ceftriaxone IV + metronidazolo IV (4 w), then amoxicillin/clavulanic acid PO (2 w)	Yes	Cure
**(7)**	74/M	LBP (18 w)	Extensively carious residual dentition	Spondylodiscitis T12-L1	No	(1) Not reported (2) *V. parvula*	Not reported	Penicillin G IV/IM (6 w)	No	Cure
**(8)**	61/F	LBP and paravertebral muscle spasm + Fever (1 w)	Rheumatoid arthritis	Spondylodiscitis L5-S1	No	(1), (2) *V. parvula*	Not reported	Ceftriaxone IV (6 w)	No	Cure
**(9)**	70/M	LPB (4 w)	No	Spondylodiscitis L3-L4	No	(1) Not performed (2) *Veillonela* spp.	Not reported	Not reported	No	Cure
**(10)**	68/M	LPB (3 w)	Bilateral sinusotomy and turbinectomy for carcinoma	Spondylodiscitis L1-L2	No	(1) *Veillonella*. spp. (2) *V. parvula*	S: penicillin, amoxicillin/clavulanic acid, cefoxitin, clindamycin, imipenem, and metronidazole.	Amoxicillin/clavulanic acid IV (2 w) Then amoxicillin/clavulanic acid PO (4 w)	Yes	Cure
**(11)**	67/M	LPB (2 w)	Dental dislocation following an accidental fall	Spondylodiscitis L1-L5	No	(1), (2) *Veillonella* spp.	S: meropenem, ampicillin/sulbactam and piperacillin/tazobactam, and borderline sensitivity to metronidazole and penicillin.	Ceftriaxone IV (6 w)	No	Cure
**(12)**	82/M	LBP + Fever (2 w)	Decompressive laminectomy at L2–S1 and C4–C5, along with anterior cervical discectomy	Discitis T10-L1	Right tibial osteomyelitis, tricuspid and mitral valve endocarditis	(1) *V. parvula* (2) not performed	S: Amoxicillin/clavulanate, Ceftriaxone, Clindamycin, Metronidazole R: Penicillin	Ceftriaxone IV then Amoxicillin/clavulanate PO (27 w)	Yes	Cure
**(13)**	72/M	LBP (7 w)	periodontitis and dental caries	Spondylodiscitis L2-L3	No	(1) Negative (2) *V. parvula*	Not reported	Ceftriaxone IV + Metronidazole IV (6 w)	No	Not reported
**(14)**	52/M	LBP (1 w)	No	Spondylitis L5/S1	No	(1), (2) *V. parvula*	Ampicillin, ceftazidime, levofloxacin	Ampicillin IV (8 w)	No	Cure
**Present case**	80/M	Neuropathic metatarsalgia (8 w)	Permanent dental prosthesis	Discitis L3-L5	No	(1) *V. parvula* (2) Not performed	S: Penicillin G, ampicillin/sulbactam, ceftriaxone, ciprofloxacin, gentamicin, vancomycin R: Piperacillin, ceftriaxone, azithromycin	Amoxicillin/clavulanic acid (6 w)	No	Cure

S: susceptibility; R: resistance; W: weeks; M: male; F: female.

## Data Availability

Data will be made available upon request.

## Abbreviations

The following abbreviations are used in this manuscript:

MRI	Magnetic resonance imaging
CT	Computed tomography
LPB	Low pain back
w	Weeks
S	Susceptibility
R	Resistance
IV	Intravenous
PO	Per os/oral administration
